# Impact of excess sugar on the whole genome DNA methylation pattern in human sperm

**DOI:** 10.1080/17501911.2024.2439782

**Published:** 2024-12-20

**Authors:** Josefine Jönsson, Alexander Perfilyev, Unn Kugelberg, Signe Skog, Axel Lindström, Sabrina Ruhrmann, Jones K. Ofori, Karl Bacos, Tina Rönn, Anita Öst, Charlotte Ling

**Affiliations:** a Epigenetics and Diabetes Unit, Department of Clinical Sciences in Malmö, Lund University Diabetes Centre, Lund University, Scania University Hospital, Malmö, Sweden; b Department of Biomedical and Clinical Sciences, Division of Cell Biology, Linköping University, Linköping, Sweden

**Keywords:** Human, sperm, epigenetics, DNA methylation, diet intervention, sugar, WGBS, DMR

## Abstract

**Aims, Patients & Methods:**

Dietary factors may regulate the epigenome. We aimed to explore whether a diet intervention, including excess sugar, affects the methylome in human sperm, and to describe the sperm methylome. We used Whole Genome Bisulfite Sequencing (WGBS) to analyze DNA methylation in sperm taken at three time points from 15 males during a diet intervention; i) at baseline, ii) after one week on a standardized diet, and iii) after an additional week on a high-sugar diet providing 150% of their estimated total energy expenditure.

**Results:**

We identified seven nominal diet-associated differentially methylated regions in sperm (*p* < 0.05). The diet was nominally associated with methylation of 143 sites linked to fertility (e.g. *AHRR*, *GNAS*, and *HDAC4*), 313 sites in imprinted genes (e.g. *GLIS3*, *PEG10*, *PEG3*, and *SNURF*), and 42 sites in top 1%-expressed genes (e.g. *CHD2*) (*p* < 0.05). In sperm, 3’UTRs and introns had the highest levels of methylation, while 5’UTRs and CpG islands had the lowest levels. Non-expressed genes in human sperm were hypomethylated in exons compared with transcribed genes.

**Conclusions:**

In human sperm, DNA methylation levels were linked to gene expression, and excess sugar had modest effects on methylation on imprinted and highly expressed genes, and genes affecting fertility.

## Plain Language Summary

Diet can influence the way genes are regulated in the body. Using a method called Whole Genome Bisulfite Sequencing, we examined changes in DNA methylation, which can affect how genes are turned on or off. Our findings showed that, in human sperm, DNA methylation was linked to how genes are expressed. In this study, we also looked more specifically at whether consuming high amounts of sugar could affect DNA methylation levels in human sperm. We found that one week of excess sugar intake had small effects on DNA methylation, also in genes that are important for fertility and in genes that are usually highly active. More research is needed to confirm these findings.

## Graphical abstract

**Figure:**
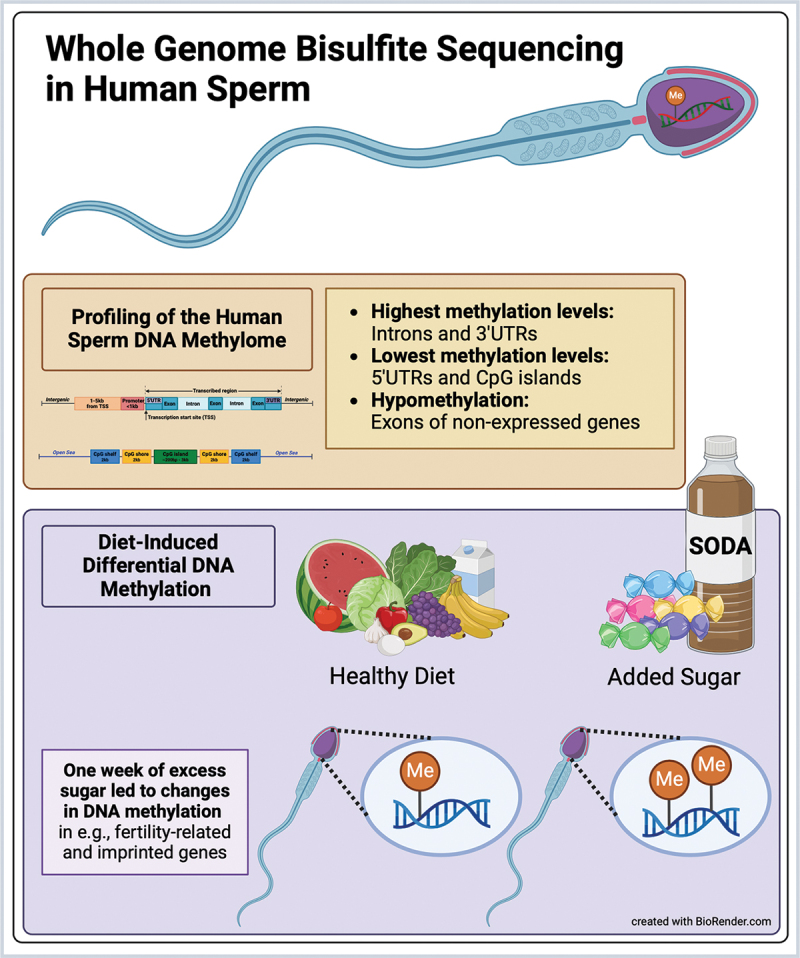

## 1. Background

In the past five decades, sperm counts have halved globally, and the rate of decline is accelerating [1]. Sperm count, motility, and morphology are all closely linked to fertility. The integrity of sperm DNA is vital for fertilization, and the majority of mammalian sperm DNA is protected by being highly condensed by protamines [2]. A smaller portion of the sperm chromatin, containing genes important for spermiogenesis and early fertilization, is histone-bound [2]. Additionally, the chromatin is organized into functional loop domains attached to the nuclear matrix, which aids the regulation of gene transcription and DNA replication [2]. Proper DNA methylation is critical for offspring viability [3] and fertility [4]. It is also an important epigenetic mechanism in processes such as embryonic development, genomic imprinting, X-chromosome inactivation, and transcriptional regulation [5]. DNA methylation occurs mainly on cytosines followed by guanine – so-called CpG-sites – and is highly affected by environmental and lifestyle factors [5–11]. Lifestyle-induced epigenetic changes may subsequently also affect human sperm quality. Indeed, the paternal diet has been shown to induce intergenerational metabolic responses through epigenetics in rodents and fruit flies [12–14]. Additionally, sncRNA levels and motility of the spermatozoa in humans are affected by the diet [15,16]. During development, mammalian cells undergo two waves of reprogramming of the DNA methylation patterns. The first wave takes place in the germline, where sex-specific methylation patterns are created at imprinted loci [17]. The second wave happens after fertilization, where the blastocyst gains its somatic DNA methylation pattern, repressing transposons, which is essential for genomic stability and germ cell function [3,18].

Sperm promoters are kept in an active chromatin state bound by transcription factors (TFs) but are transcriptionally inactive [19]. This may be due to the presence of protamines on sperm DNA, preventing the transcription machinery from transcribing [19]. The TF-bound sites may be blocked from the second wave of DNA methylation reprogramming, and potentially, these sites remain accessible to the transcription machinery later in development [19]. As environmental factors can influence the distribution of transcription factors, this constitutes a possible mechanism for how the epigenetic pattern is transmitted between and thereby persists over generations [19]. The heritability of DNA methylation levels in blood was estimated to be nearly 20% [20], suggesting that an altered DNA methylome might be transmitted to the next generation. Additionally, while the human sperm methylome was homogenous and hypomethylated, the sex chromosomes were the most hypomethylated [21]. Although some studies have analyzed the methylome in the human sperm [22,23] and some have related methylation to expression using 450k arrays and qPCR [24], to our knowledge, it remains unknown whether the sperm’s DNA methylation pattern is influenced by a diet including an excess of added sugars and whether the global sperm methylome is linked to expression levels of the transcriptome.

Whole Genome Bisulfite Sequencing (WGBS), which is an advantaged method compared to array-based methods because it allows for single-base resolution, genome-wide coverage, and reduced bias regarding probes and cross-hybridization, was used to **I)** investigate whether a diet intervention, including added sugar in the form of sweets and sweetened drinks to reach 150% of the recommended daily intake (RDI), affected DNA methylation in sperm from 15 men, and **II)** dissect and describe the human sperm DNA methylome and relate it to gene expression.

## 2. Research design and methods

### 2.1. Study cohort

The study was approved under the Declaration of Helsinki by the regional ethical board at Linköping University, Sweden (permit number: 2016/183–31). Written informed consent was obtained from all participants. The diet intervention included 15 men aged 20–27 with a BMI below 30 kg/m^2^, recruited by advertising at the University of Linköping, Sweden (Table 1), and was originally reported in [15]. The inclusion criteria were having an age between 20 and 30 years old, not having obesity (i.e., body mass index (BMI) <30.0 kg/m^2^, https://www.who.int/health-topics/obesity#tab=tab_1), being nonsmoker, and omnivore. For the two weeks of diet intervention, the recruited participants committed to only consuming meals provided by the research team, refraining from alcohol, and were asked to keep their physical activity levels constant. The initial cohort comprised 16 men; however, one participant did not complete the study. To maintain consistency with the data presented in the original article describing the cohort [15] we have preserved the sample numbers, accordingly, retaining participants 1–2 and 4–16 for comparative purposes.

**Table 1.** Characteristics of participants (*n* = 15). Values in bold indicate statistical significance with *p* < 0.05.

	Mean ± SD (range)				Wilcoxon signed rank test with continuity correction		
Characteristic	Baseline	Healthy	Added Sugar	Groupwise comparisons (*p-*value)	Baseline vs Healthy (*p-*value)	Baseline vs Added Sugar (*p-*value)	Healthy vs Added Sugar (*p-*value)
Age (years)	23.27 ± 2.28 (21.50–25.00)	23.27 ± 2.28 (21.50–25.00)	23.27 ± 2.28 (21.50–25.00)	1	NA	NA	NA
Height (m)	1.84 ± 0.08 (1.77–1.88)	1.84 ± 0.08 (1.77–1.88)	1.84 ± 0.08 (1.77–1.88)	1	NA	NA	NA
Weight (kg)	75.83 ± 10.23 (68.23–81.80)	75.25 ± 9.86 (68.54–80.83)	76.70 ± 9.92 (70.39–83.13)	0.923	0.06372	**0.02557**	**0.0008545**
BMI (kg/m^2^)	22.48 ± 2.57 (20.31–24.37)	22.31 ± 2.39 (20.17–24.31)	22.73 ± 2.32 (20.92–24.42)	0.892	0.06372	**0.02557**	**0.00116**
Sperm concentration(x10^6^/ml)*	32.40 ± 25.25 (11.00–44.00)	32.20 ± 20.18 (22.00–44.00)	28.87 ± 18.27 (14.00–39.50)	0.88	1	0.2712	0.2712
Progressive motilesperm (%)*	47.93 ± 14.36 (33.50–60.50)	53.00 ± 13.04 (49.00–61.00)	55.93 ± 8.63 (52.50–63.00)	0.207	0.6947	**0.003687**	0.2781
Total motile sperm (%)*	53.00 ± 14.10 (40.00–65.00)	58.00 ± 13.11 (54.50–66.00)	60.87 ± 8.07 (57.00–67.50)	0.207	0.6098	**0.004607**	0.3276
Fat mass (kg)**	11.53 ± 4.08 (9.11–12.84)	11.71 ± 3.76 (9.22–12.92)	11.86 ± 4.12 (9.77–13.31)	0.975	0.8647	0.2442	0.8469
Fat free mass (kg)	64.30 ± 8.06 (59.93–70.02)	63.55 ± 7.61 (58.89–68.80)	64.85 ± 7.99 (61.20–71.33)	0.903	**0.03015**	0.06372	**0.0004272**
Plasma cholesterol (mmol/L)*	4.59 ± 1.15 (3.80–5.20)	4.38 ± 1.38 (3.70–4.65)	4.46 ± 1.31 (3.85–4.70)	0.906	0.06371	0.1727	0.3096
Serum triglycerides(mmol/L)**	1.20 ± 0.81 (0.84–1.30)	1.08 ± 0.63 (0.66–1.10)	2.13 ± 1.28 (1.40–2.55)	**0.008**	0.268	**0.001329**	**0.001782**
Plasma HDL cholesterol (mmol/L)*	1.55 ± 0.42 (1.25–1.75)	1.49 ± 0.41 (1.20–1.60)	1.36 ± 0.38 (1.10–1.55)	0.423	0.2311	**0.01187**	0.05481
Serum LDL cholesterol (mmol/L)*	2.48 ± 0.87 (1.80–2.80)	2.38 ± 1.18 (1.85–2.35)	1.90 ± 0.54 (1.45–2.15)	0.202	0.1614	**0.01182**	0.09595
Plasma non-HDL cholesterol (mmol/L)*	3.02 ± 1.17 (2.20–3.30)	2.87 ± 1.44 (2.25–2.85)	3.08 ± 1.40 (2.50–3.20)	0.91	0.09571	0.9164	**0.01418**
Fasting plasma glucose (mmol/L)*	5.13 ± 0.33 (4.90–5.30)	5.09 ± 0.29 (4.90–5.20)	5.15 ± 0.38 (4.90–5.35)	0.877	0.5061	0.9164	0.5994

*BMI, body mass index; HDL, High-density lipoprotein;* *kg, kilogram; LDL, low-density lipoprotein;* *m, meter; SD, standard deviation*.

* = Warning messages.

1: In wilcox.test.default(A$Concentration_sperm_x106_p_ml:

B$Concentration_sperm_x106_p_ml.

cannot compute exact p-value with ties.

2: In wilcox.test.default(A$Concentration_sperm_x106_p_ml:

B$Concentration_sperm_x106_p_ml.

cannot compute exact p-value with zeroes.

** = Warning message.

In wilcox.test.default(A$Per_fat_mass__kg, B$Per_fat_mass__kg, paired = TRUE):

cannot compute exact p-value with ties.

### 2.2. Diet intervention

**Figure 1.**
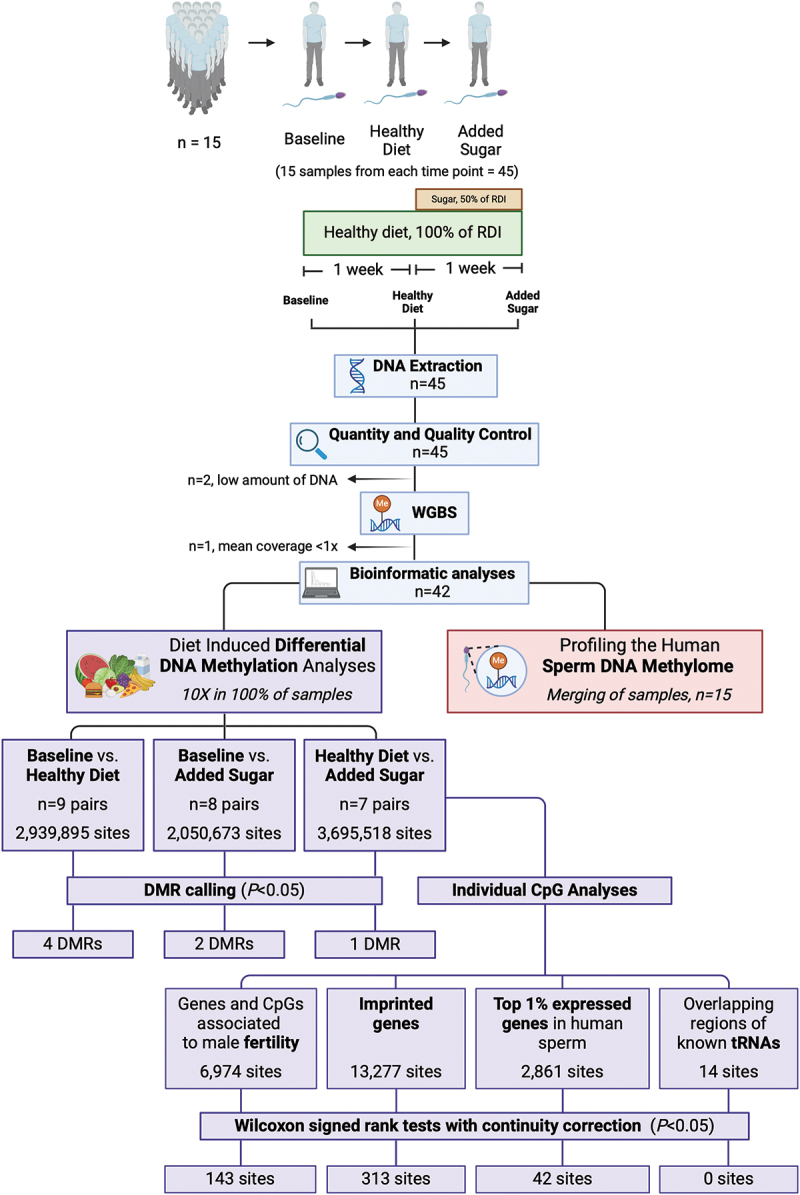
Study design and workflow from participant inclusion to whole genome bisulfite sequencing (WGBS) and statistical analyses. Fifteen participants received a standard healthy diet for one week, equivalent to 100% of their recommended daily intake (RDI), calculated based on their basal metabolic rate (BMR) and total energy expenditure (TEE), followed by one week of added sugar, the equivalent of 50% of their RDI. Sperm samples were collected from all men at three time points: i) at Baseline, ii) after one week of the healthy diet, and iii) after one week of the added sugar diet. Created with BioRender.com. *BMR, basal metabolic rate; DMR, differentially methylated region, RDI, recommended daily intake; TEE, total energy expenditure*.

The participants were subjected to a 2-week diet intervention. The first week involved a standardized healthy diet based on the Nordic Nutrition Recommendations [25] with an energy intake corresponding to the study participant’s estimated total energy expenditure (TEE) (Figure 1). The estimated TEE for each participant was assumed to correspond to their individual daily energy requirement (i.e., the energy intake required to maintain their current body weight). The diet had the subsequent energy distribution: breakfast 25%, lunch 30%, dinner 30%, and snacks 15%. The research team provided all meals, and the participants were instructed to consume only the provided food and refrain from any additional intake. During the first week of the study, the participants were instructed to only drink water. During the second week, the same standardized healthy diet was provided to the participants with the addition of sweetened drinks and sweets (not including licorice or chocolate, due to the impact on blood pressure and high-fat content, respectively), corresponding to 50% of their estimated TEE. The proportion of sweets and sweetened beverages was adjusted according to the participant’s preferences to increase compliance. The intake of sweets, sweetened drinks, and the standardized healthy diet resulted in an energy intake of 150% of their estimated TEE. Detailed information on enrollment, diet intervention, and clinical measurements has been reported elsewhere [15].

### 2.3. Human semen collection

Semen samples were obtained by masturbation after two to three days of sexual abstinence from each study participant (*n* = 15) at three time points: i) before the diet intervention (Baseline), ii) after one week of a healthy diet (Healthy Diet), and iii) after the diet rich in sugar (Added Sugar) (Figure 1). Samples were collected between November 2016 and October 2017 at the Reproductive Medicine Center, University Hospital of Linköping, in sterile 50 ml non-spermiotoxic polypropylene tubes and liquefied at room temperature. Motile spermatozoa were prepared using a discontinuous (1.5 ml 80%/1.5 ml 40%) PureSperm (Nidacon Int, Gothenburg, Sweden) gradient. Neat semen (0.5–2.0 ml) was layered on the top of the gradient and centrifuged at 300 g for 20 minutes, followed by resuspension of the pellet in equilibrated sperm preparation media (PureSperm Wash; Nidacon). The sperm suspension was thereafter centrifuged at 500 g for 10 minutes and resuspended and diluted to appropriate concentration/volume motile sperm (using a Makler counting chamber; Cellvision, Heerhugowaard, the Netherlands) in equilibrated PureSperm Wash. After preparation, the proportion of motile spermatozoa was > 97% for all samples. After the liquefaction of semen, standard semen parameters were obtained according to World Health Organization criteria [26].

### 2.4. DNA extraction

Ethanol precipitation was used to extract DNA from the interphase and organic (phenol) phase of semen samples lysed in QIAzol Lysis Reagent (Qiagen, Hilden, Germany) (please see [15] for RNA extraction). The DNA pellet was washed with sodium citrate and ethanol and redissolved in 8 mm NaOH. The DNA sample was pH neutralized by adding 0.1 M HEPES and 0.1 M EDTA to a final concentration of 1 mM.

### 2.5. WGBS analyses

Quantity and quality control (QC) of the 45 DNA samples were done using the Femto Pulse System (Agilent, Santa Clara, CA, USA). Two samples were excluded from the study due to low amount of DNA (<2 ng) (Figure 1). Genomic DNA (2.2–50 ng) was sheared to 400–500 bp using the Covaris E220 System (Covaris, LLC., Woburn, MA, USA) and treated with sodium bisulfite using the EZ DNA Methylation Gold kit (Zymo Research Corporation, Irvine, CA, USA) according to the manufacturer’s instructions. Sequencing libraries were prepared for 43 samples using the SPlinted Ligation Adapter Tagging (SPLAT) protocol [27] (Table S1). Cluster generation and paired-end 150 bp read length sequencing were performed using S4 flow cells with the NovaSeq6000 system and v1.5 sequencing chemistry (150 cycles, Illumina Inc., San Diego, CA, USA).

### 2.6. WGBS analysis pipeline

The sequencing data was processed using R [28]. QC was done before and after paired-end trimming using FastQC [29] and MultiQC [30]. Paired-end reads were trimmed using Trim Galore! [31]. Genome indexing was done using Genome Reference Consortium Human Build 38 (hg38) using an R annotation package [32]. Sequencing reads were aligned, and count data was generated using Bismark [33]. Reads and alignment statistics before and after deduplication and sequencing depth are reported in Tables S1 and S2.

### 2.7. DMR calling

**Figure 2.**
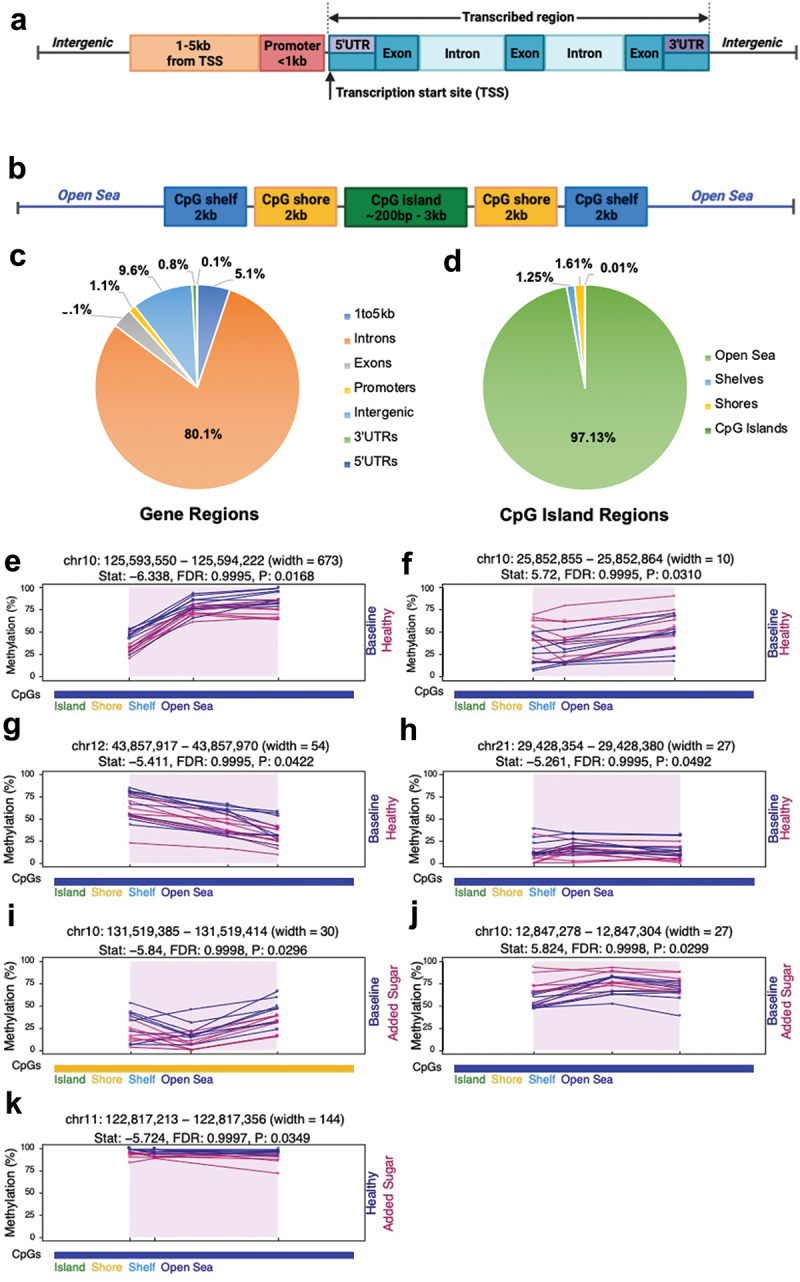
CpG-sites are mapped to (a) Gene regions based on functional genome distribution, where the promoter is defined as the region between the TSS and 1 kb upstream, (b) and to CpG island regions based on CpG content. A CpG island is defined as a 200 bp long (or longer) stretch of DNA with a CG content of 50% and an observed CpG/expected CpG in excess of 0.6, the shore is the flanking region of CpG islands, 0–2000 bp. The shelf is the region’s flanking island shores, i.e., covering 2000–4000 bp distant from the CpG island, and the Open Sea is everything else. (c) Proportion of analyzed CpG-sites, with ≥ 10X CpG coverage in all samples, of different gene regions; 5.1% (748,684 annotations) in regions 1 to 5kb from transcription start site (TSS), 80.1% (11,650,021 annotations) in introns, 3.1% (456,772 annotations) in Exons, 1.1% (166,760 annotations) in promoters, 9.6% (1,395,278 annotations) in Intergenic regions, 0.8% (109,539 annotations) in 3’ untranslated regions (UTRs), and 0.1% (12,865 annotations) in 5’ UTRs, and (d) The proportion of analyzed CpG-sites in different CpG island regions; 97.13% (3,589,443 sites) in Open Sea, 1.25% (46,346 sites) shelves, 1.61% (59,328 sites) in shores, 0.01% (401 sites) in CpG islands. Diet-associated differently methylated regions (DMRs) (*p* < 0.05, *n* = 15 per diet). (e-h) compared to the baseline, the healthy diet leads to hypomethylation of three different DMRs and hypermethylation at one DMR. The lines represent individual smoothed methylation level estimates for baseline (blue) and healthy diet (magenta). (i-j) compared to baseline, the added sugar diet leads to hypomethylation of one DMR and hypermethylation of one DMR. The lines represent individual smoothed methylation level estimates for baseline (blue) and added sugar (magenta). (k) Compared to the healthy diet, the added sugar diet leads to hypomethylation of one DMR. The lines represent individual smoothed methylation level estimates for healthy diet (blue) and added sugar (magenta). All are intergenic, and regarding CpG island regions, all but one are in the Open Sea. The dots represent the methylation level estimates of an individual CpG, and each dot’s size represents coverage. CpG and genic annotation tracks are shown below each plot. A-B are created with BioRender.com. *TSS, transcription start Site; 5’UTR, 5’ untranslated Region; 3’UTR, 3’ untranslated Region; kb, kilobase pairs; bp, base pairs.*

To identify diet-associated differentially methylated regions (DMRs), we applied dmrseq [34], which, using transformed methylation proportions, fits a linear regression model using generalized least squares with a nested autoregressive correlated error structure. This provides efficient and stable estimation procedures for the time points/diets tested (Baseline versus Healthy Diet, Baseline versus Added Sugar, and Healthy Diet versus Added Sugar). The dmrseq package [34] “weighs” samples based on coverage and methylation state, meaning a CpG-site with 3 out of 3 reads methylated is not treated the same as a CpG with 50 out of 50 reads methylated. i.e., a higher coverage allows for more certainty of the methylation state. For the DMR calling, we required ≥ 10× CpG coverage (sequencing depth) of individual CpG-sites in all samples, a minimum DMR length of three CpGs with a cutoff of 0.05 (≥5% difference in methylation between groups), and 10 permutations. DMR annotation to gene and CpG island regions was done using hg38 and annotatr [35] (Figure 2(a–d)).

### 2.8. Gene expression in human sperm

Publicly available human sperm RNA-sequencing data for 12 subjects [36] was downloaded from the NCBI SRA database (https://www.ncbi.nlm.nih.gov/sra, Accession: PRJNA573604). These subjects are distinct from the 15 individuals for whom other data are reported in this article. Data preparation and adapter trimming were performed using Trim Galore! [31]. QC was done using FastQC [29] and MultiQC [30] tools. Downstream analysis was done using R [28]. No outliers were detected, and the RNA-sequencing data for all 12 samples were merged for further analyses. Transcript quantification of the merged samples was done using Salmon [37] and DESeq2 [38]. Genes were annotated to hg38. Expression levels were divided into four groups, with the non-expressed genes having < 2 mean normalized counts (*n* = 20,791), and the remaining expressed genes (≥2 mean normalized counts) were divided into three groups of similar size, categorized into low- (*n* = 4,151), medium- (*n* = 4,171), and high-expressed (*n* = 4,295) genes. Friedman rank sum test was used to find associations between the mean DNA methylation levels and the four expression levels in different genomic regions.

### 2.9. Identifying individual differently methylated CpG-sites

To test if the Added Sugar diet versus the Healthy Diet impacted the degree of DNA methylation of individual CpG-sites, we included CpG-sites with ≥ 10× CpG coverage in all samples, resulting in 3,695,518 CpG-sites annotated to 22,705 unique genes (Figure S1). Due to the limited statistical power resulting from the sample size, a pre-selection of CpG-sites was performed, thereby reducing the number of tests requiring correction for multiple tests (Figure 1). The pre-selection included individual **I)** CpG-sites and genes that are vital in sperm quality and male fertility based on the review from Åsenius *et al*. [39] (Table S3), resulting in 6,974 CpG-sites (Table S4), **II)** CpG-sites annotated to 129 imprinted genes (https://www.geneimprint.com/site/genes-by-species.Homo+sapiens.imprinted-All, accessed on 14 December 2023) (Table S5) resulting in 13,227 CpG-sites annotated to 92 out of the 129 genes (Table S6), **III)** CpG-sites annotated to the top 1% expressed protein-coding genes (159 genes) in human sperm [36] (Table S7) resulting in 2,861 CpG-sites (Table S8), and **IV)** CpG-sites positioned at the exact same coordinates as known tRNAs (Table S9), resulting in 14 CpG-sites (Table S10). CpG-sites annotated to tRNAs were studied based on the impact of excess sugar on their expression in human sperm in our previous study [15].

DNA methylation data for these CpG-sites were extracted from our human sperm data.

For analysis **I)** we extracted CpG-sites directly using Illumina CpG cluster IDs or using the gene symbol for CpG-sites annotated to genes linked to sperm quality and fertility [39]. The online “Lift Genome Annotations” (https://genome.ucsc.edu/cgi-bin/hgLiftOver) tool was used to convert older assemblies to the latest (hg38) to harmonize the data across different genome versions and get accurate genomic locations of methylation sites. When retrieving genomic location from the Illumina HumanMethylation27 Product Support Files (HumanMethylation27 Content List, EXCEL file, https://support.illumina.com/downloads/humanmethylation27_product_support_files.html, downloaded on 21 November 2023) four methylation sites were not found; cg07719512, cg08296824, cg04856685, cg24133080. Additionally, methylation sites cg00705255 and cg04807108 could not be converted to hg38. For analysis **II)**, the gene symbol was used to extract CpG-sites. For *INPP5F* V2, we removed “V2” and the “at” symbol present in the gene list for *SNORD115@* was removed before extracting methylation sites from our dataset. The “at” symbol is used to indicate gene clusters. For analysis **III)**, the gene symbol was used to extract CpG-sites. Lastly, in analysis **IV)**, the genomic coordinates for known tRNAs were used to extract CpG-sites. Wilcoxon signed rank tests with continuity correction were used in all four analyses to study the impact of added sugar (compared to the Healthy Diet) on DNA methylation of individual CpG-sites.

## 3. Results

### 3.1. WGBS in human sperm

WGBS was performed to analyze DNA methylation at base-pair resolution in the sperm from 15 men (Figure 1). Characteristics of the participants are reported in Table 1. After QC, we profiled genome-wide DNA methylation at 26.9 million sites in the CG context with an average sequencing depth of 10× in the sperm samples. Alignment statistics and sequencing information are reported in Tables S1 and S2. An average of 238,336,623 reads were mapped to the human reference genome (hg38) after deduplication. The purity of the sperm samples was then evaluated by first analyzing the DNA methylation levels for some CpG sites annotated to *XIST* and *DDX4*, two genes previously shown to have hypomethylated regions in normal sperm [40–42]. The sperm samples included in this study had low methylation levels of these CpG sites (Table S11). We then utilized the fact that small RNA sequencing had been performed on all the sperm samples, to examine whether they share similarity to potential contaminants in ejaculated seminal samples, namely epithelial cells and leukocytes. The small RNA profiles of our sperm samples [15] were compared with profiles generated on other human sperm samples [43], epithelial cells [44], and leukocytes [45]. This analysis showed that the small RNA libraries from all our samples are “sperm-like,” with the expected high tsRNA content [46], and suggested little risk of contamination of leukocytes and epithelial tissue (Figure S2).

### 3.2. Diet-associated DMRs in human sperm

We investigated the effect of diet on the DNA methylome in sperm based on DMRs identified by dmrseq [34]. DMR calling was done on 2.9, 2.1, and 3.7 million cytosines in the CG context with an average sequencing depth of 11.7×, 12.5×, and 11.7× for Baseline versus Healthy, Baseline versus Added Sugar, and Healthy versus Added Sugar, respectively (Table S1). Requiring three or more consecutive differentially methylated sites based on nominal *P*-values (*p* < 0.05), we found seven diet-associated DMRs. Four DMRs for Baseline versus Healthy Diet, two DMRs for Baseline versus Added Sugar, and one DMR for Healthy Diet versus Added Sugar, all annotated to intergenic regions and with a DMR length of three CpG-sites (Table 2 and Figure 2(e–k)).

**Table 2.** Differentially methylated regions (DMRs) in human sperm in response to different diets (baseline, healthy diet, and added sugar) *p* < 0.05.

*Annotations to DMRs*										Diets tested
DMR chromosomal position	DMR width (bp)	Number of CpGs in DMR	Sum of smoothed beta values (DNA methylation)	Β-coefficient values	Test statistic	Permutation *P*-value	*q*-value	CpG Island region	Gene region	
chr10:125593550–125594222	673	3	0.3417	−0.3019	−6.3383	0.0168	0.9995	Open Sea	Intergenic	Baseline vs. Healthy Diet
chr10:25852855–25852864	10	3	0.4344	0.3245	5.7203	0.0310	0.9995	Open Sea	Intergenic	Baseline vs. Healthy Diet
chr12:43857917–43857970	54	3	0.3722	−0.2406	−5.4113	0.0422	0.9995	Open Sea	Intergenic	Baseline vs. Healthy Diet
chr21:29428354–29428380	27	3	0.1848	−0.2652	−5.2614	0.0492	0.9995	Open Sea	Intergenic	Baseline vs. Healthy Diet
chr10:131519385–131519414	30	3	0.3869	−0.3546	−5.8398	0.0296	0.9998	Shore	Intergenic	Baseline vs. Added Sugar
chr10:12847278–12847304	27	3	0.2726	0.2611	5.8240	0.0299	0.9998	Open Sea	Intergenic	Baseline vs. Added Sugar
chr11:122817213–122817356	144	3	0.2040	−0.3070	−5.7241	0.0349	0.9997	Open Sea	Intergenic	Healthy Diet vs. Added Sugar

Chromosomal positions are based on hg38.

Baseline vs. Healthy Diet *n* = 9, Baseline vs. Added Sugar *n* = 8, Healthy Diet vs. Added Sugar *n* = 7. *DMR, differently methylated region*.

### 3.3. Impact of the diet intervention on methylation of individual CpG-sites and the global methylome

**Figure 3.**
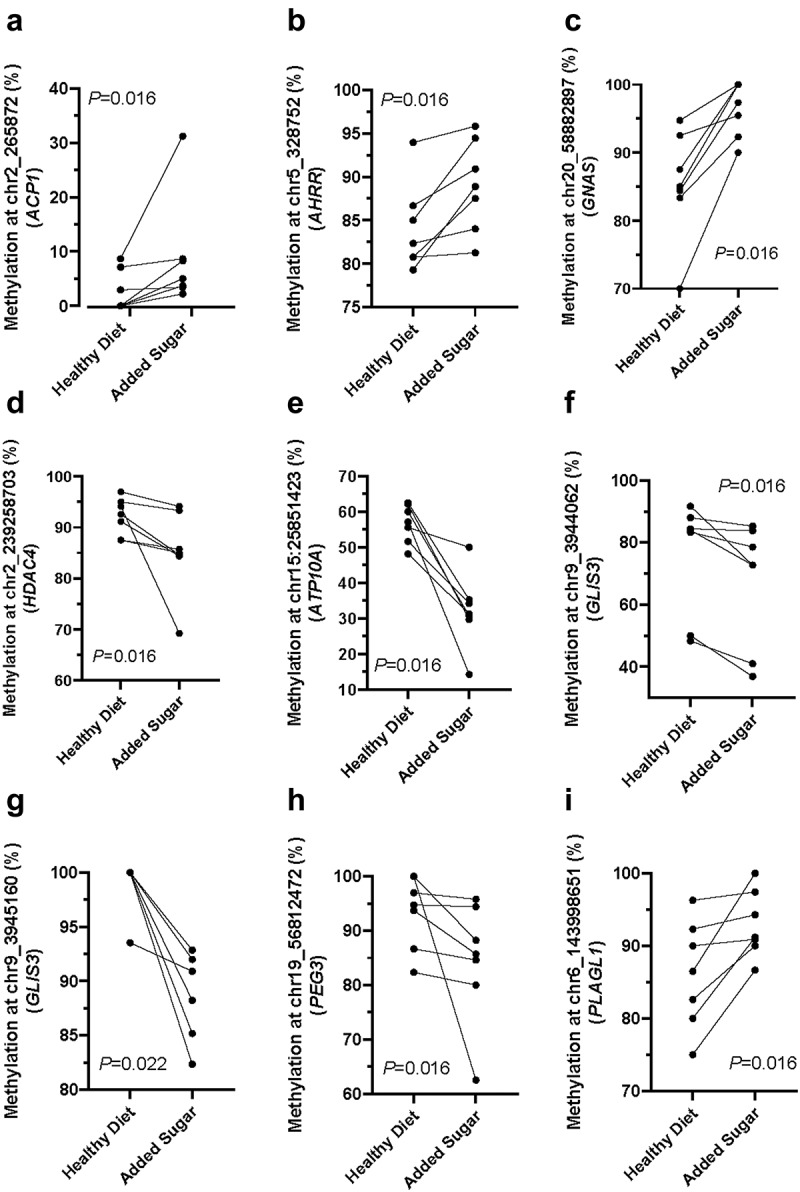
Diet-associated different DNA methylation of individuals CpG-sites based on nominal *P*-values (*p* < 0.05), requiring ≥ 10 reads for included sites and samples, as well as DNA methylation going in the same direction in all sperm samples when comparing the healthy diet versus added sugar. DNA methylation of CpG-sites annotated to (a) *ACP1*, (b) *AHRR*, (c) *GNAS* showing increased methylation, (d) *HDAC4*, (e) *ATP10A*, (f-g) *GLIS3*, (h) *PEG3* showing decreased methylation, and I) *PLAGL1* showing increased methylation after the added sugar versus the healthy diet in all sperm samples with ≥ 10 reads in the site (*n* = 7 for all panels except (f), *n* = 6). Data was analyzed using Wilcoxon signed rank tests with continuity correction to study the impact of added sugar compared to the healthy diet on DNA methylation of individual CpG-sites.

We further investigated the impact of the Healthy Diet versus Added Sugar on DNA methylation of **I**) CpG-sites and genes with known function in male fertility [39], **II**) CpG-sites annotated to 129 imprinted genes (https://www.geneimprint.com/site/genes-by-species.Homo+sapiens.imprinted-All), **III**) CpG-sites annotated to the top 1% (159) expressed protein-coding genes in human sperm [36], or **IV**) CpG-sites at coordinates of known tRNAs. We chose to study methylation of CpG sites annotated to the top 1% expressed protein-coding genes and tRNAs because DNA methylation is known to regulate gene expression [5] and because we previously showed the diet-intervention altered expression of tRNAs in sperm [15]. For all four analyses, we required a coverage of ≥ 10 reads in all samples taken during the Healthy Diet and Added Sugar diet for included CpG-sites. Based on nominal *P*-values (*p* < 0.05), the Added Sugar diet affected the DNA methylation of **I**) 143 of 6,974 analyzed sites annotated to 54 genes with known function in male fertility (Table S4), **II**) 313 of 13,227 analyzed sites annotated to 81 imprinted genes (Table S6 and Figure 3), **III**) 42 of 2,861 analyzed sites annotated to 28 of the top 1% expressed protein-coding genes in human sperm (Table S8), and **IV**) none of the 14 analyzed sites based on exact genomic coordinates as known tRNAs (Table S10). The sites showing diet-associated methylation changes are annotated to genes with known function in male fertility included *ACP1*, *AHRR*, *DUSP22*, *GNAS*, *HDAC4*, *KCNJ16*, *PAX8, PLAGL1*, *PTPRN2*, and *SNURF* (Table S4 and Figure 3), and the top 1% expressed genes included *CHD2* and *CPEB2* (Table S8 and Figure 3). Additionally, the following genes, *GNAS, LOC101927932, PEG3, PEG3-AS1, PLAG1, PWRN1, SNHG14, SNRPN, SNURF, TNRC18*, and *ZIM2*, which showed diet-associated changes in DNA methylation, belong to at least two of the following groups that were studied; **I**) genes with known function in male fertility, II) imprinted genes, or **III**) top 1% expressed protein-coding genes in human sperm (Table S4 , S6 and S10). Overall, this analysis found that the Added Sugar diet nominally affected the DNA methylation level of 486 individual sites (Table S4 , S6, and S8). Notably, numerous of these sites showed diet-associated changes in DNA methylation that went in the same direction in all analyzed samples (Figures 3 and S3). However, due to the modest number of sperm samples and the large number of statistical tests these results did not stand correction for multiple testing.

**Figure 4.**
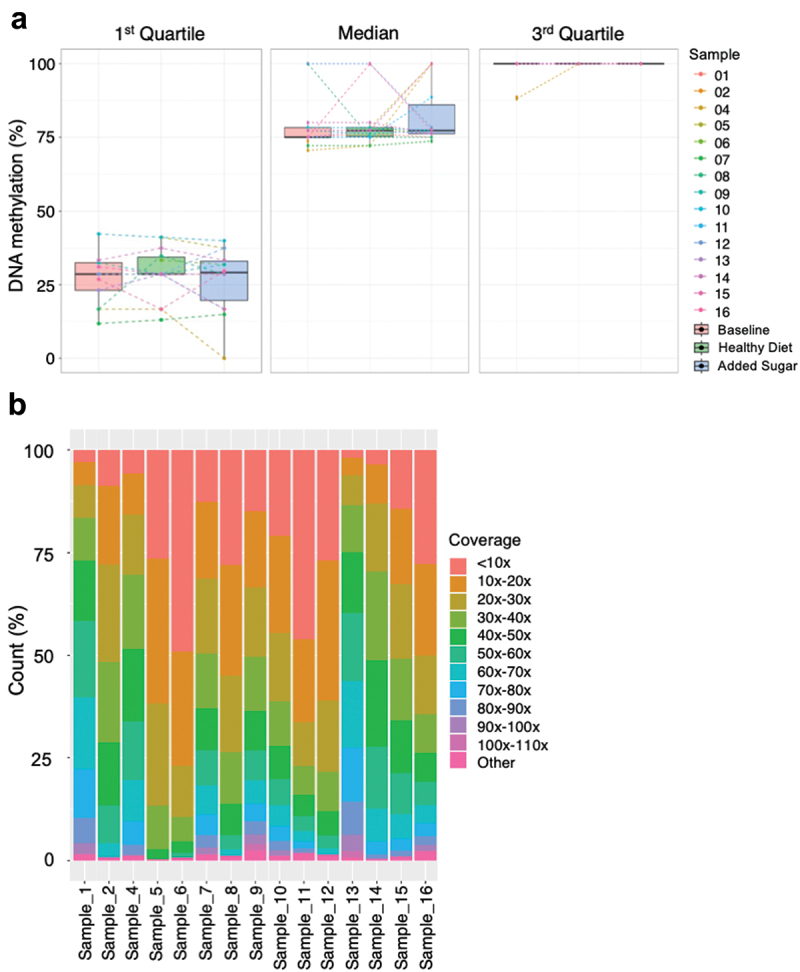
(a) Boxplot visualizing the variability between individuals and diets based on the global degree of methylation. The methylation levels for 22,780,770 CpG-sites in each sample with a mean coverage of ≥ 10× (*n* = 41) were used to calculate the 1^st^, 2^nd^, and 3^rd^ quartile. The 1^st^ quartile represents the CpG-sites with the lowest methylation level (lowest 25%), 2^nd^ quartile represents the median methylation level, and 3^rd^ quartile represents the CpG-sites with the highest methylation level (highest 25%) (b) Coverage proportion for the whole genome bisulfite sequencing data in sperm for each individual after merging data from the three different time points.

We next investigated the impact of the three diets on the “*global*” methylome in human sperm by calculating the 1^st^, 2^nd^, and 3^rd^ quartiles per diet (Baseline, Healthy Diet, and Added Sugar). The quartiles were calculated based on the methylation levels for 22,780,770 CpG-sites (the number of overlapping CpG-sites for all three time points) in each sample with a mean coverage of ≥ 10× (*n* = 41). The 1^st^ quartile represents the CpG-sites with the lowest methylation level (lowest 25%), 2^nd^ quartile represents the median methylation level, and 3^rd^ quartile represents the CpG-sites with the highest methylation level (highest 25%) (Figure 4(a)). The diet intervention did not impact the “*global*” methylome in human sperm, and the “*global*” DNA methylation pattern at the three different time points in each of the 15 men did not differ. Additionally, the Added Sugar diet had no significant effect on the variance for the “*global*” degree of methylation in any of the quartiles (*p* = 0.33 and *p* = 0.81 for the variance in Healthy Diet versus Added Sugar in the 1^st^ and 2^nd^ quartiles, respectively. The sample variance was too small to do a statistical test for the 3^rd^ quartile, where the methylation value was 100% for 39 out of 41 samples) (Figure 4(a)).

### 3.4. Profiling the human sperm DNA methylome

We proceeded to characterize the overall variability of the sperm methylome in the 15 men included in this study. Since the effects of the diet intervention on the global methylome were modest (Figure 4(a)), we merged the methylation data of the samples from the three time points of the intervention for each individual to increase the sequencing depth and CpG coverage of the genome (Figure 4(b)). This resulted in an average CpG coverage of 28.7 (Table S2).

**Figure 5.**
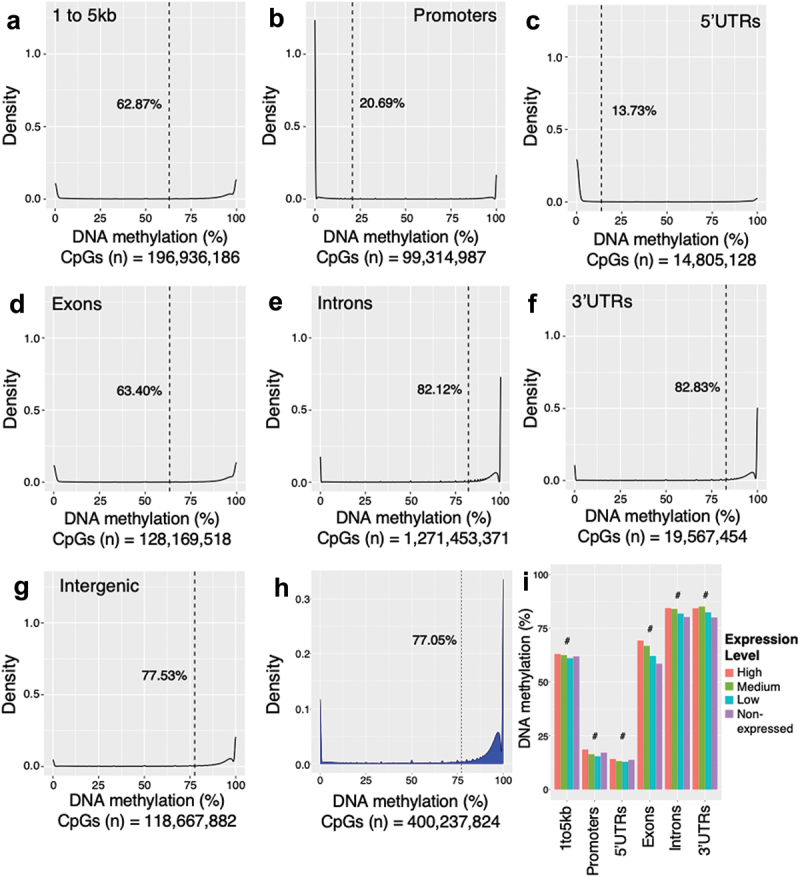
Density plots showing the degree of DNA methylation in the human sperm samples (*n* = 15) in different gene regions (a–g), Overall DNA methylation distribution (h) and average DNA methylation levels in different gene regions baded on expression levels (i) Using the merged WGBS data including all three time points/diets. (a) the region of 1 to 5 kb upstream of the TSS had a mean DNA methylation degree of 62.87%. (b) Promoters had a mean DNA methylation degree of 20.69%. (c) 5’UTRs had a mean DNA methylation degree of 13.73%. (d) Exons had a mean DNA methylation degree of 63.40%. (e) introns had a mean DNA methylation degree of 82.12%. (f) 3’UTRs had a mean DNA methylation degree of 82.83%. And (g) Intergenic regions had a mean DNA methylation degree of 77.53%. (h) Density plot of the overall methylome in human sperm (*n* = 15). Peaks at around 0 and around 100% methylation. The mean methylation degree was 77.05%. (i) average DNA methylation levels, using the WGBS data, in different gene regions of non-expressed genes and expressed genes divided into high-, medium-, and high-expressed. Friedman rank sum test was used to test for differences in the average DNA methylation levels between the four groups (high-, medium-, low- and non-expressed genes) for the different gene regions (#<0.05, as analyzed by Friedman rank sum tests, *p* = 9.3×10^−10^ for 1-5kb, *p* = 1.6×10^−9^ for promoters, *p* = 9.3×10^−10^ for 5’UTR, *p* = 9.3×10^−10^ for exons, *p* = 9.3×10^−10^ for introns, and *p* = 2.6×10^−9^ for 3’UTR). Results from Dunn’s multiple comparison test can be seen in table S12. CpGs (n) is the total number of CpGs analyzed in all samples in each specific region. *WGBS, whole genome bisulfite sequencing; TSS, transcription start Site; 5’UTR, 5’ untranslated Region; 3’UTR, 3’ untranslated Region; kb, kilobase pairs; bp, base pairs.*

The distribution and degree of DNA methylation vary with different genomic regions and CG density (Figure 2(a–d)); for example, the gene body was previously found to have the highest degree of methylation and promoter regions the lowest in human pancreatic islets [47]. Here, we used dmrseq [34] to generate density plots visualizing the overall distributions of the methylation levels through different genomic regions in human sperm (Figures 5(a–g) and 2(a)). We found the introns and 3’UTRs to have the highest degree of DNA methylation, with an average of around 82% (Figure 5(e,f)), while the lowest level was in the 5’UTRs, with an average of 13.7% methylation (Figure 5(c)). Additionally, we found the highest degree of methylation in the CpG island shelves with an average of 87% methylation and the lowest degree in CpG islands with an average degree of methylation of 8.5% (Figures S4A-C and 2(b)). We then plotted the overall distribution of DNA methylation in human sperm, which is bimodal, with the first peak at around 0%, representing CpG-sites with low levels of methylation, and the second peak at about 100%, displaying that the majority of CpG-sites are highly/fully methylated with an average degree of 77.05% methylation (Figure 5(h)).

#### 3.4.1. Characteristics of methylated cytosines in the CG, CHG, and CHH context in human sperm

Cytosine DNA methylation can be divided into three types: CG, CHG, and CHH (H = A, C or T). Since the biological role of methylation in non-CG context has been debated, we investigated the degree of DNA methylation of cytosines in these different contexts. The average DNA methylation levels of cytosines in the CHG and CHH contexts were very low at 0.37% ± 0.03% and 0.35% ± 0.04%, respectively, compared with 77.05% as seen in the CG context (Figure 5(h)), hence suggesting CG-methylation to be the most important in human sperm.

#### 3.4.2. DNA methylation levels of genes categorized as non-, low-, medium and high-expressed in human sperm

Next, we explored the relationship between DNA methylation and gene expression levels using WGBS from the sperm of the 15 men included in the diet intervention and publicly available RNA-seq data from the sperm of 12 men [36]. A total of 33,408 genes were identified in the RNA-seq data, of which *n* = 20,791 genes (62%) were categorized into non-expressed (<2 mean normalized counts). The remaining 12,617 genes were identified as expressed and were divided into three bins categorized as low- (*n* = 4,151), medium- (*n* = 4,171), and high-expressed (*n* = 4,295) genes. We then studied the degree of DNA methylation in different gene regions and the four expression levels (Figure 5(i)). Using the Friedman rank sum test, we found associations between the mean DNA methylation levels and the four expression levels in different genomic regions (Figure 5(i)). For example, non-expressed genes in human sperm had lower methylation levels in exon regions compared with the transcribed genes based on the Dunns’ post hoc test (Figure 5(i) and Table S12). This is consistent with our previous study in somatic tissues, e.g., human pancreatic islets [47], but, to our knowledge, it has not been studied in human sperm.

#### 3.4.3. Gene expression of imprinted genes in human sperm

We finally studied the expression levels of 129 known imprinted genes, of which 79 are maternally imprinted, 39 are paternally imprinted, and 11 are isoform-dependent, random, or not indicated, in human sperm (https://www.geneimprint.com/site/genes-by-species.Homo+sapiens.imprinted-All, accessed on 14 December 2023). Genomic imprinting is the process where DNA methylation suppresses one gene copy, allowing only one copy of a gene to be expressed in an individual. Which gene copy is expressed depends on the gene. We found 76 of the 129 imprinted genes to be expressed in human sperm; 31 were highly expressed, e.g., *PEG3* and *DNMT1* – encoding the enzyme responsible for maintaining DNA methylation, 26 were medium expressed, e.g., *GLIS3* – an important transcription factor in islet beta-cells [48], and *PLAGL1*, and 19 were low expressed, e.g., *INS* – the gene encoding insulin, and *LIN28B* (Figure S5A and Table S13). Additionally, Figure S5B-C presents the distribution of the expression of maternally and paternally imprinted genes, respectively.

## 4. Discussion

This study used WGBS of human sperm to dissect the methylome and characterize changes in DNA methylation after a short diet intervention rich in sugar. While the Added Sugar diet had limited effects on the global methylome and DMRs, we found nominal diet-associated changes in DNA methylation of individual CpG-sites of genes previously linked to male fertility [39], paternally expressed genes, and genes with high expression in human sperm [36]. When dissecting the human sperm methylome, we found the 3’UTRs and introns to have the highest methylation levels (≈82%), while the 5’UTRs and CpG islands had the lowest methylation levels (≈14% and ≈ 8.5%, respectively). Additionally, non-expressed genes in human sperm were hypomethylated in exons compared with transcribed genes.

The prevalence of obesity and related metabolic diseases are increasing at alarming rates worldwide due to an overconsumption of unhealthy diets rich in free sugar and saturated fat, urbanization, and reduced physical activity [49]. Importantly, high sugar intake in men is also associated with lower sperm concentration [50] and motility [51]. We and others have shown that diets and physical activity can influence the epigenome, e.g., DNA methylation levels of specific CpG-sites, as well as gene expression in human tissues such as adipose tissue and skeletal muscle [6,7,9,52–56]. Recently, we also showed that a healthy lifestyle, including physical activity and a low-energy Mediterranean diet, during pregnancy in mothers with obesity was associated with altered DNA methylation levels in the cord-blood of the offspring [10]. Epigenetic and transcriptomic changes may further affect cell metabolism and function and subsequently influence disease development [57].

Additionally, some studies have investigated the impact of different diets and physical exercise on the DNA methylation pattern in human and rodent sperm [39,58–60]. However, to our knowledge, the effect of added sugar by overconsumption of sweets and sweetened drinks on the DNA methylation pattern in human sperm has not been studied. Interestingly, Nätt *et al*. showed that human sperm are sensitive to a diet high in sugar [15], with sperm motility and tsRNA levels impacted by added sugar. In the present study, we investigated the genome-wide DNA methylation pattern in the sperm samples from the diet intervention performed by Nätt *et al*. [15]. When correcting for multiple testing, neither the global methylome nor DMRs changed in sperm after adding excess sugar to the diet. By our definition, DMRs require differences in DNA methylation of three or more consecutive CpG-sites, and we found seven diet-associated DMRs based on nominal *P*-values, and three of these were linked to the Added Sugar diet. Our study can be considered a pilot study as the number of statistical tests performed was extensive due to the large number of CpG-sites analyzed by WGBS, while the number of samples was modest. Due to the limited statistical power, we tested whether there was an impact of the Added Sugar diet on DNA methylation of a selection of individual CpG-sites for candidate genes previously linked to male fertility [39], being annotated to imprinted genes, the top 1% genes with highest expression in human sperm [36], or known tRNAs. The Added Sugar diet affected DNA methylation of 486 individual sites annotated to 151 unique genes with nominal *P*-values. Notably, the methylation level of numerous of these sites changed in the same direction in sperm from all individuals when excess sugar was added, suggesting a true effect. For example, in all sperm samples analyzed for individual CpG-sites in the present study, the Added Sugar diet increased DNA methylation of *ACP1*, *AHRR*, *GNAS*, and *PTPRN2* and decreased methylation of a site in *HDAC4*. *ACP1* encodes a phosphotyrosine protein phosphatase, and carriers of genetic polymorphism in this gene, representing ≈ 10% of the population, have lower spermatic concentrations and more atypical spermatozoa [61]. Eight of the 151 unique genes (i.e., *ANK2*, *DSCAM*, *GLIS3*, *MAGI2*, *NTM*, *PPP1R9A*, *SGCE*, and *ZFHX3*) have more than 10 CpG-sites (ranging from 11 to 101 sites) shown to be affected by the Added Sugar diet with *MAGI2* being the most impacted. Moreover, DNA methylation levels of *AHRR*, encoding Aromatic Hydrocarbon Receptor Repressor, in blood is a well-established biomarker of smoking [62,63], and smoking in men is associated with reduced sperm quality [64]. Interestingly, cigarette smoke condensate activated the aryl hydrocarbon receptor (Ahr), leading to the upregulation of antioxidant enzymes in rodent spermatocytes and altering the growth pattern of spermatocytes in vitro via Ahr-Nrf2 signaling [65]. *GNAS* is in a locus with a complex imprinted expression pattern, and differential DNA methylation of this gene has previously been linked to abnormal semen, including low sperm count and mobility [66]. In addition, *GNAS* methylation in sperm was inversely correlated with sperm concentration and associated with follicle-stimulating hormone and luteinizing hormone levels [67]. *HDAC4* encodes an epigenetic enzyme that deacetylates histones. While the Added Sugar diet resulted in reduced DNA methylation of *HDAC4* in sperm in the present study, we have previously shown that regular exercise increased DNA methylation and decreased expression levels of *HDAC4* in adipose tissue [54], demonstrating how unhealthy and healthy lifestyles can regulate the epigenome of this gene.

Genomic imprinting – the silencing of genes by DNA methylation – is a process controlled by whether the gene is inherited from the father or mother [68]. Some paternally derived germline DNA methylation imprints have been identified, and after fertilization, DMRs can also arise at imprinted regions [68]. Imprinted control regions can retain methylation during post-fertilization epigenetic reprogramming, unlike the rest of the gametically methylated regions, which are demethylated after fertilization. For these reasons, we were interested in investigating the impact of the Added Sugar diet on DNA methylation in imprinted genes in sperm. Several paternally imprinted genes, including *ATP10A*, *CPA4*, *HOXA4*, *MAGI2*, *NTM*, *PPP1R9A*, *SLC22A3*, and *UBE3A*, have sites that showed changes in DNA methylation in sperm going in the same direction from all individuals after one week of overconsumption of sugar. The same holds true for several maternally imprinted genes, including *GLIS3*, *LIN28B*, *PEG3*, and *PLAGL1*. Of note, a deficiency in the gene product from the paternally imprinted gene *ATP10A*, a phospholipid flippase, has been found to cause male-specific infertility in mice, which is believed to be partly due to lower sperm motility and reduced sperm count [69]. Additionally, *MAGI2*, a gene where we found > 100 sites affected by the Added Sugar diet, exhibits differently methylated sites in both sperm and blood when comparing infertile patients and fertile controls [70,71]. *GLIS3*, a maternally imprinted gene, is a candidate gene for diabetes, and we have previously shown that palmitate exposure altered the expression and DNA methylation of *GLIS3* in human pancreatic islets [72]. Interestingly, we also found that diabetes-associated genetic variants overlap with open chromatin of *GLIS3* as well as of several other candidate genes for type 2 diabetes genes such as *TCF7L2* and *KCNQ1* [73] and based on the strong interaction between genetic variation and DNA methylation, and the potential impact of non-genetic factors [74–76], we chose not to filter our CpG-SNPs in the present study. Additionally, altered methylation in the sperm of some of these maternally imprinted genes, e.g., *PLAGL1*, has been linked to male fertility [39], while *PEG3* belongs to the top 1% expressed genes in human sperm [36].

RNA was discovered in sperm already in the 1980s, and since then, numerous studies have investigated the sperm transcriptome and its biological role [15,36]. Recently, Corral-Vazquez used RNA-sequencing to analyze the transcriptome in sperm from 12 men [36]. Based on these data, they defined 12,853 mRNAs as expressed in human sperm and classified 5,100 as highly expressed. DNA methylation was initially thought to silence expression, and functional studies, using, for example, luciferase assays, have shown that increased DNA methylation in promoter and enhancer regions decreases transcriptional activity in autosomal cells [57,77,78]. However, today, it is well established that the impact of DNA methylation on gene transcription is more complex. For example, hypermethylation has been observed in exons of expressed versus non-expressed genes in DNA extracted from human pancreatic islets [47] and in human B-lymphocytes [79]. Hence, in the present study, we further tested if the degree of DNA methylation in different genomic regions was linked to the gene expression level in human sperm by first dividing the transcriptome into non-, low-, medium, and high-expressed genes. Interestingly, we found a clear link between the degree of methylation and gene expression within the genes, where the exons of non-expressed genes had lower methylation than expressed genes in human sperm.

There are some strengths and limitations related to this study. One limitation is the modest number of participants in this study, which, together with the large number of tests performed, makes it difficult to find significant diet-associated changes in DNA methylation after correction for multiple testing. Even if some sites changed the degree of methylation in the same direction in sperm from all participants after the Added Sugar diet, these did not remain significant based on FDR. We acknowledge the importance of exercising caution and recognizing the potential for false positives when assessing targeted analyses since this approach focuses on a limited portion of the genome. Perhaps even more so when assessing imprinted loci as they often exhibit tissue-specific expression patterns, further complicating their identification and validation. Hence, future larger studies need to confirm our findings. Additionally, several of the diet-associated changes in DNA methylation were modest and may subsequently have a limited biological function. Of note, for some sites that seem to change with diet, the degree of methylation seen in the control samples and the changes vary quite a lot. Sperm samples from a larger number of men are needed to further evaluate how much methylation varies in these sites. Another limitation includes the low amount of sperm DNA from some participants, making it difficult to sequence these samples deep throughout the genome, thereby reducing the possibility of detecting minor diet-induced differences in methylation. When investigating diet-associated changes in DNA methylation of individual CpG-sites, we subsequently only included sites with more than 10 reads in all included samples. Overall, we cannot rule out that these factors, at least in part, explain why we do not find any DMRs on FDR < 5%. Additionally, the diet intervention was short, only two weeks, whereas the process of human spermatogenesis is estimated to be 42–76 days [80]. The transit and maturation process in epididymis is, however, much shorter. In humans, it can be as short as 2–6 days [81]. Interestingly, recent work in mice, where the transit time of sperm in epididymis is around 10–13 days, identified a two-week diet-intervention to be the most efficient time to generate intergenerational metabolic effects [82]. Additionally, we found effects of the two-week diet-intervention on the sperm sncRNA levels and spermatozoa motility (see ref [15] and [16]). Our previous studies showed that a five-day high fat diet as well as 36-h fasting could alter the levels of DNA methylation in both human skeletal muscle and adipose tissue, supporting that shorter diet interventions can induce DNA methylation changes in humans [6,53,56,83,84]. However, it is possible, based on the importance of proper DNA methylation in sperm, that the DNA methylation pattern already formed in sperm will be moderately affected by a two week change in diet. Changes in DNA methylation are likely to occur during spermatogenesis, for example, during loss of methylation in meiosis or the remethylation immediately afterward. Additionally, human meiosis requires ~2 weeks, spermiogenesis takes ~3 weeks, mature sperm DNA is in a near-crystalline form, and there are limited amounts of TET or DNMTs in the sperm for large remodeling of the methylome during the travel of sperm through the genital tract. Therefore, to see stronger effects on the DNA methylation pattern, the dietary intervention should potentially be extended over the lifespan of the sperm to ensure all sperms have formed during the intervention.

The strengths of this study are the controlled diet, which is also provided to the participants, the strict inclusion criteria, and the homogeneity of participants regarding age and BMI. Additionally, concerns have been raised regarding somatic DNA contamination; we implemented density gradient separation, a measure shown to mitigate the potential for cellular contamination in the semen samples [85].

## 5. Conclusion

In conclusion, this study provides a comprehensive picture of the DNA methylation pattern in human sperm. It also demonstrates the link between sperm methylation and RNA levels, as well as the importance of epigenetic regulation of different genomic regions. Our results further support that one week of a sugar-rich diet does not impact the global sperm methylome. However, our data suggest that Added Sugar may change the methylation levels of individual CpG-sites in genes linked to male fertility and paternally expressed genes, but these data need to be validated in future studies.

## Supplementary Material

Supplemental Material

## Data Availability

The sperm DNA methylation dataset generated for this study was deposited in the LUDC repository (https://www.ludc.lu.se/resources/repository, accession number: LUDC2024.02.1). Summary and meta data are available upon request through https://www.ludc.lu.se/resources/repository. However, individual-level WGBS data from the human sperms are not publicly available due to ethical and legal restrictions related to the Swedish Biobanks in Medical Care Act, the Personal Data Act and European Union’s General Data Protection Regulation and Data Protection Act. The sperm RNA-sequencing dataset is available at https://www.ncbi.nlm.nih.gov/sra (accession number: PRJNA573604).

## Article highlights

**Aim**

- Investigate if a diet intervention including excess sugar affected DNA methylation in human sperm
- Profile the DNA methylome of the human sperm

**Methods**

- 15 participants were subjected to a 2-week diet intervention, with and without added sugar
- Samples were collected before the diet intervention (Baseline), after one week of a healthy diet (Healthy Diet), and after the diet rich in sugar (Added Sugar)
- Whole Genome Bisulfite Sequencing was performed and used to investigate diet-associated DNA methylation patterns and profile the DNA methylome

**Diet-Associated DMRs in Human Sperm**

- Seven nominally diet-associated differently methylated regions (DMRs)

**Impact of the diet intervention on methylation of individual CpG-sites and the global methylome**

- Multiple nominally associated methylation sites related to, e.g., fertility and imprinted genes

**Profiling the Human Sperm DNA Methylome**

- The overall distribution of DNA methylation in human sperm is bimodal, with peaks around 0% and 100%, and an average methylation level of 77%
- Highest methylation levels in introns and 3‘UTRs
- Lowest methylation levels in 5‘UTRs and CpG islands
- Hypomethylation in exons of non-expressed genes compared to transcribed genes

**Conclusion**

- This study provides a comprehensive picture of the DNA methylation pattern in human sperm. Demonstrating the link between sperm methylation and RNA levels, as well as the importance of epigenetic regulation of different genomic regions. Our results support that one week of excess sugar had modest effects on the DNA methylome and methylation of individual CpG-sites in genes linked to male fertility and imprinted genes
