# Supplementary figures and images for: Impact of excess sugar on the whole genome DNA methylation pattern in human sperm
Source: Epigenomics. 2024 Dec 20;17(2):89–104. doi: 10.1080/17501911.2024.2439782 (PMC11792836; doi:10.1080/17501911.2024.2439782)

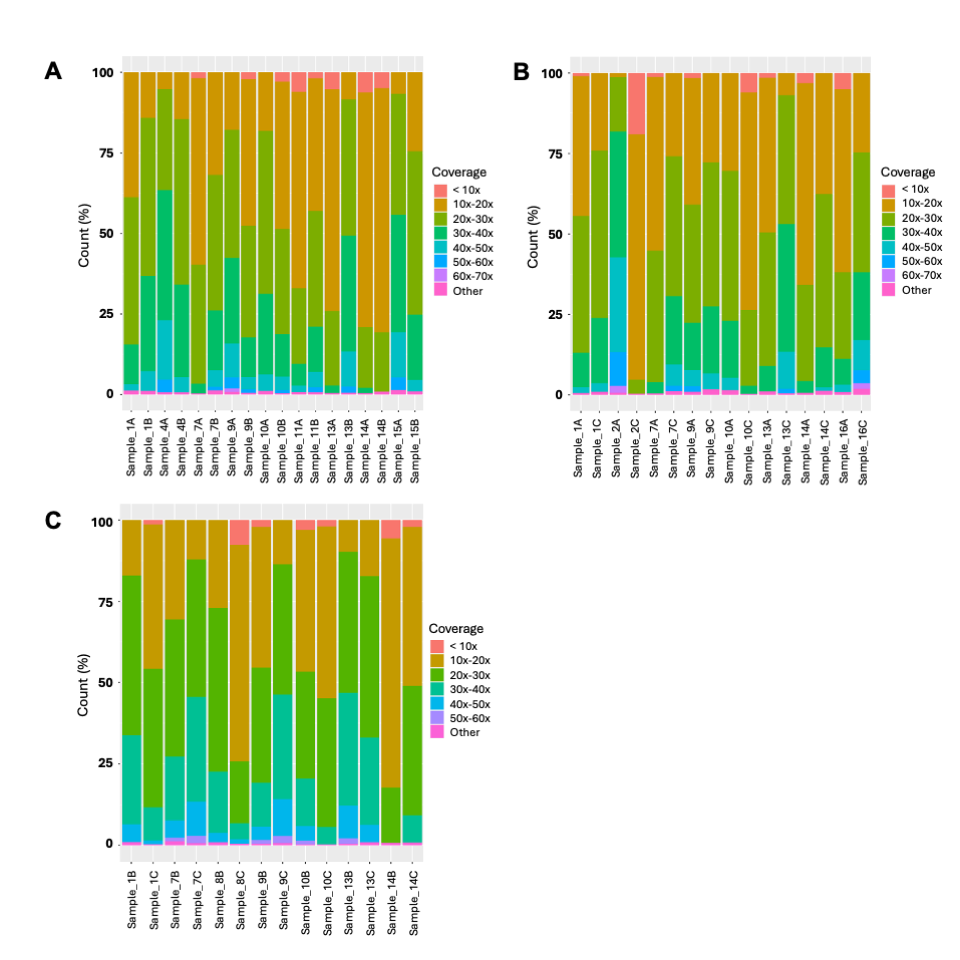

Supplement: Supplemental Material [file IEPI_A_2439782_SM2945.zip › suppl_data/FigureS1_coverage-timepoints.tiff]

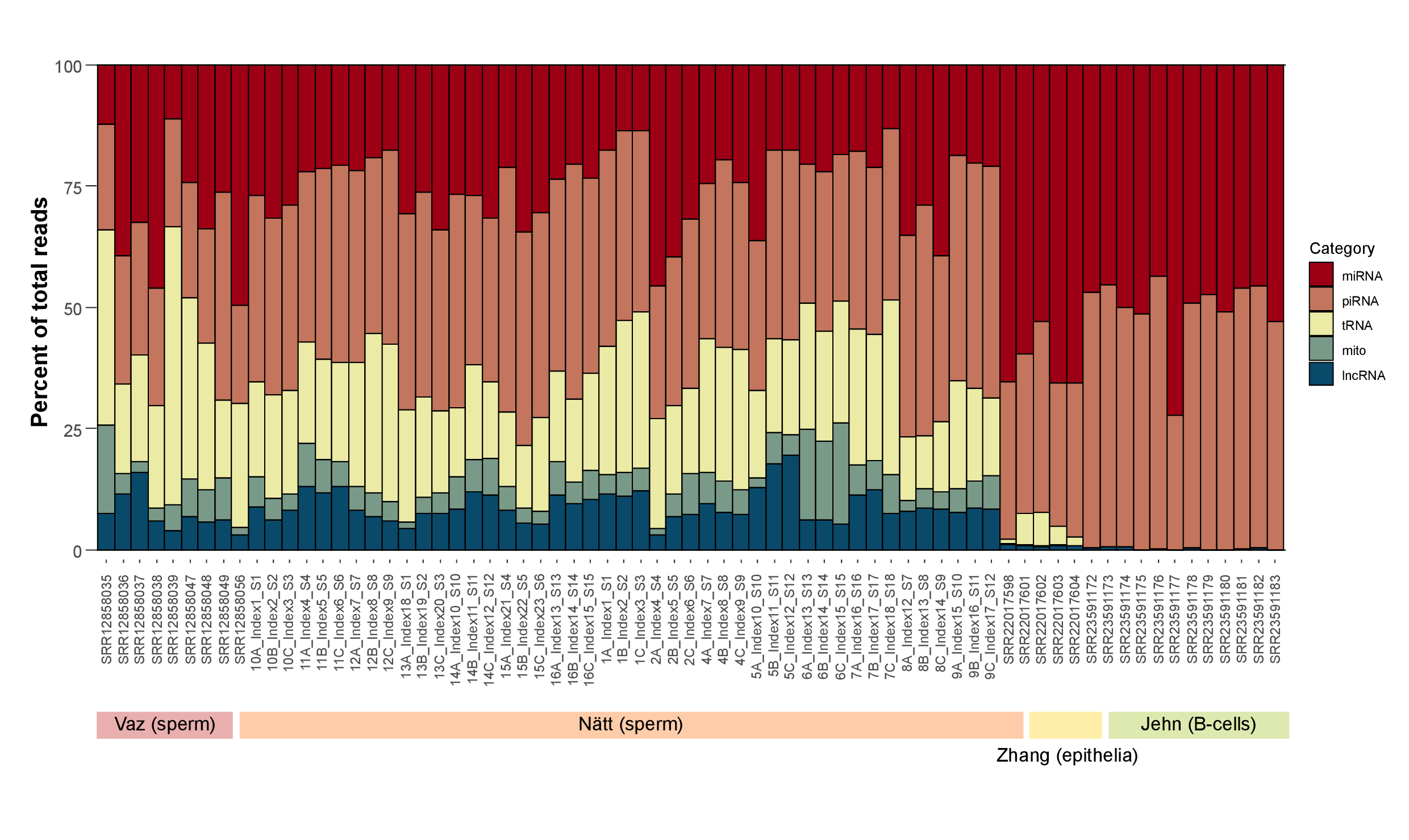

Supplement: Supplemental Material [file IEPI_A_2439782_SM2945.zip › suppl_data/FigureS2 subm.tiff]

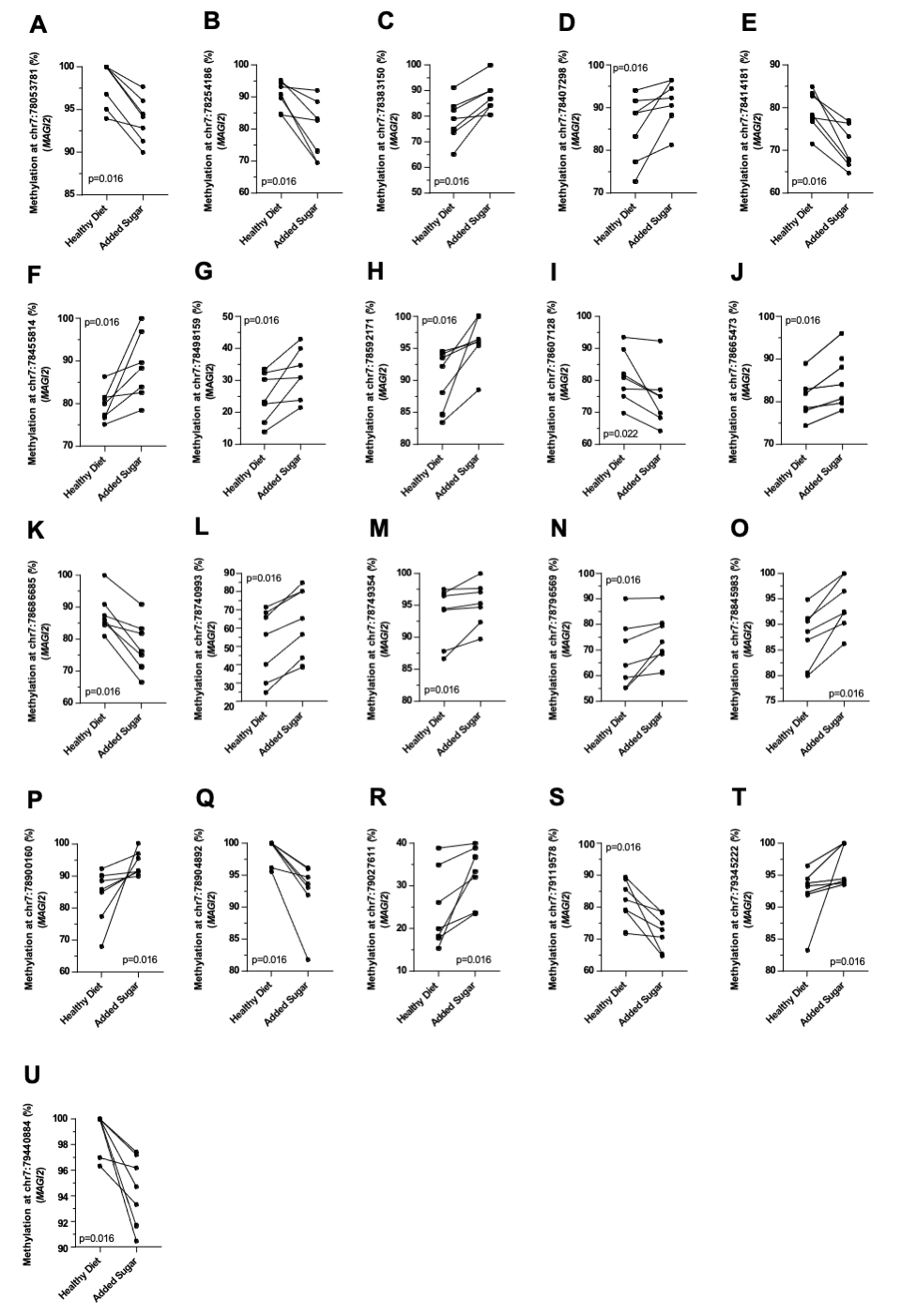

Supplement: Supplemental Material [file IEPI_A_2439782_SM2945.zip › suppl_data/FigureS3_DNAmethIndCpGs.tiff]

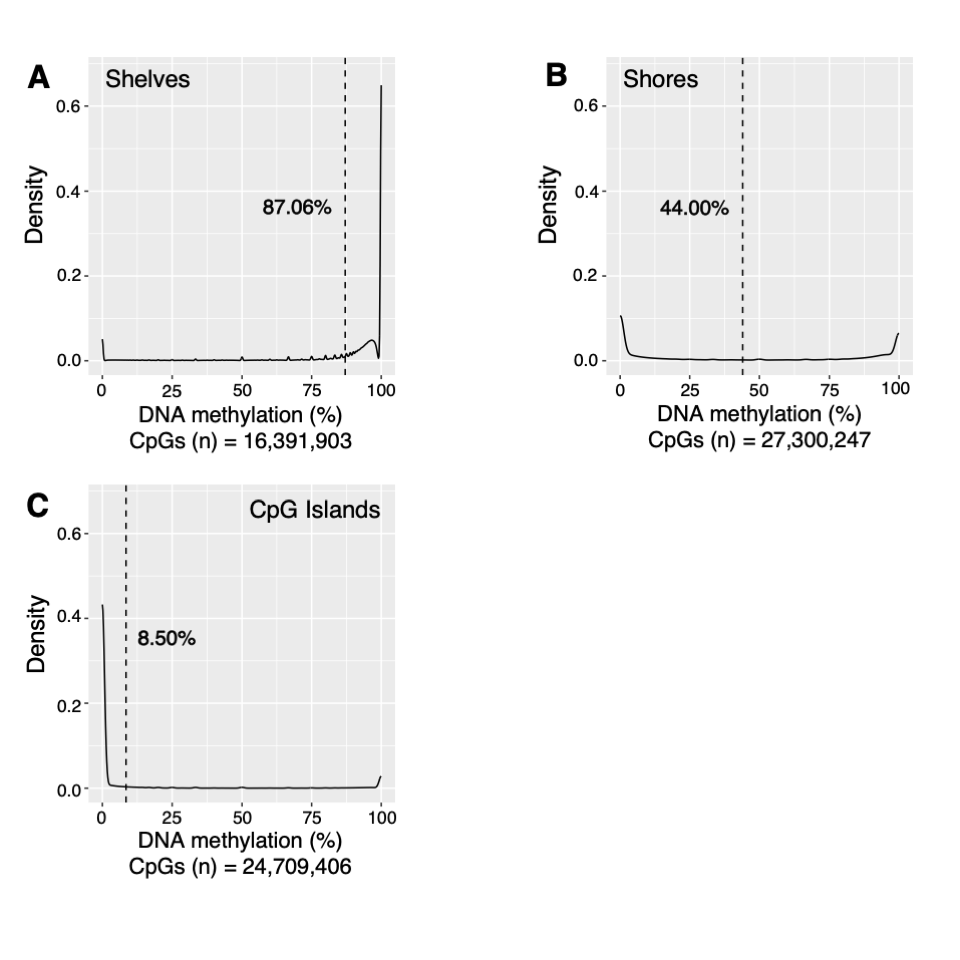

Supplement: Supplemental Material [file IEPI_A_2439782_SM2945.zip › suppl_data/FigureS4_DNAmethCpGIslandRegions.tiff]

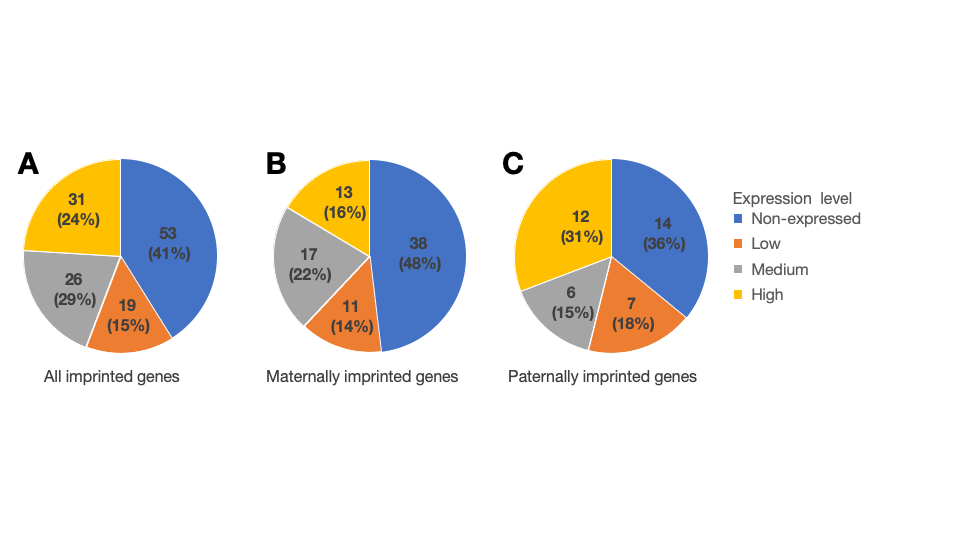

Supplement: Supplemental Material [file IEPI_A_2439782_SM2945.zip › suppl_data/FigureS5_ImprintedGenes.tiff]
